# Utilization of Educational Videos to Improve Communication and Discharge Instructions

**DOI:** 10.5811/westjem.2021.1.48968

**Published:** 2021-04-27

**Authors:** Alisa Wray, Ronald Goubert, Rishi Gadepally, Megan Boysen-Osborn, Warren Wiechmann, Shannon Toohey

**Affiliations:** University of California Irvine, Department of Emergency Medicine, Irvine, California

## Abstract

**Introduction:**

When discharging a patient from the emergency department (ED), it is crucial to make sure that they understand their disposition and aftercare instructions. However, numerous factors make it difficult to ensure that patients understand their next steps. Our objective was to determine whether patient understanding of ED discharge and aftercare instructions could be improved through instructional videos in addition to standard written discharge instructions.

**Methods:**

This was a prospective pre- and post-intervention study conducted at a single-center, academic tertiary care ED. Patients presenting with the five selective chief complaints (closed head injury, vaginal bleeding, laceration care, splint care, and upper respiratory infection) were given questionnaires after their discharge instructions to test comprehension. Once video discharge instructions were implemented, patients received standard discharge instructions in addition to video discharge instructions and were given the same questionnaire. A total of 120 patients were enrolled in each group.

**Results:**

There were significantly better survey scores after video discharge instructions (VDI) vs standard discharge instructions (SDI) for the closed head injury (27% SDI vs 46% VDI, P = 0.003); upper respiratory infection (28% SDI vs 64% VDI; P < 0.0001); and vaginal bleeding in early pregnancy groups (20% SDI vs 60% VDI, P < 0.0001). There were no significant differences in survey scores between the splint care (53% SDI vs 66% VDI; P = 0.08) and suture care groups (29% SDI vs 31% VDI; P = 0.40).

**Conclusion:**

Video discharge instructions supplementing standard written instructions can help improve patient comprehension and information retention. This better understanding of aftercare instructions is essential to patient follow-up and has been shown to improve patient outcomes.

## INTRODUCTION

Discharge of patients from the emergency department (ED) generally includes physician discussion with the patient regarding results, treatment, and discharge plan, followed by the patient receiving paper discharge instructions, which are reviewed with the patient by the nurse at the time of discharge. Although this process is standard in many EDs across the United States, it is severely flawed. Many factors including time constraints and the unpredictability of the ED contribute to this flaw.^1^ Due to time constraints on both physicians and nurses, the amount of time to answer questions and ensure patient understanding during discharge is limited. Previous research has shown that only about 45–50% of patients or caregivers are able to understand their standard discharge instructions (SDI).^2^ This is confirmed by several studies showing poor patient understanding using questionnaires at or after discharge.^3^–^5^ Decreased understanding of discharge instructions then leads to decreased compliance, inadequate follow-up, increased readmission rates, and decreased patient satisfaction.^1^ It is also known that low health literacy is associated with higher utilization of the ED and with higher rates of return within 14 days.^6^ This issue is complicated by the fact that approximately 14.5% of individuals aged 16 years or older are illiterate (National Assessment of Adult Literacy).^7^

Many recommendations have been made on how to improve patient understanding at discharge. One way that has been proposed is the use of video discharge instructions (VDI).^4^ This provides patients with a multimodal way of receiving their diagnosis, management, and treatment plan. In addition, it provides a standardization of information regardless of patients’ literacy, provider’s time to answer questions, and time constraints of nurses. Video discharge instructions have been previously shown to be effective at improving patient and caregiver understanding of their diagnosis in pediatric EDs with common complaints such as fever or closed head injury.^8^,^9^

## METHODS

This was a prospective pre- and post-intervention study with convenience sampling done at a single-center, academic tertiary care ED. The study included any adult patients discharged from the ED with any of the five selected discharge diagnoses: vaginal bleeding in early pregnancy; concussion; splint care; laceration care; or upper respiratory tract infection. Topics were selected based on the most common diagnoses seen in the ED. Five diagnoses from the top 10 most common diagnoses were selected by an expert panel of emergency physicians based on available resources and topics that were more complex and would best benefit from VDIs.

We calculated a power analysis assuming the standard deviation (SD) of correct responses to be 25%, and 100 patients in each of the two groups would provide an 80% power to detect a difference of 10% of correct responses on the questionnaires. We aimed to recruit 240 patients to allow for up to 20% incomplete data.

Questionnaires (Supplement A) were developed based on existing standard written discharge instructions in our electronic health record (EHR) system (Epic Systems Corporation, Verona, WI). These questionnaires covered common management of the diagnoses, aftercare, and return precautions. They were developed by a panel of experts (emergency physicians), and then refined by review with patients of various education levels to ensure adequate understanding.

From July 2017–November 2017 120 patients were enrolled and completed the questionnaires before implementation of VDIs. During this time, VDIs were created based on the same content in the standard written discharge instructions in the EHR system (Supplement B). Scripts and storyboards were created, edited by the same expert panel that created the questionnaire, and then reviewed by patients of various education levels to ensure appropriate level of language and understanding. Videos were then created by whiteboard video animator wizMotions (Whiteboard Studios LLC, Toronto, ON, Canada). Before videos were finalized, they were again presented to patients of various education levels to confirm appropriate level of language and understanding.

**Population Health Research Capsule**

What do we already know about this issue?

*Previous research has shown that only about 45–50% of patients or caregivers are able to understand their discharge instructions (SDI).*

What was the research question?

*To determine whether patient understanding of ED discharge and aftercare instructions could be improved through instructional videos in addition to standard written discharge instructions.*

What was the major finding of the study?

*Video discharge instructions supplementing standard written instructions can help improve patient comprehension and information retention.*

How does this improve population health?

*This better understanding of aftercare instructions is essential to patient follow-up and has been shown to improve patient outcomes.*

Once VDIs were implemented, patients received SDIs in addition to the VDIs. From February 2018–April 2018 we enrolled 120 patients in the post-intervention study. Participants were then given the same questionnaire. Questionnaires were scored and entered into an Excel spreadsheet (Microsoft Corp, Redmond, WA) to calculate the mean. We calculated statistical significance between the SDI group vs those who received VDIs using unpaired t-tests.

## RESULTS

A total of 120 patients received the SDIs and 120 patients received the VDIs. Of those patients, 42 received the splint care instructions (18 SDI, 24 VDI); 59 received suture care instructions (31 SDI, 28 VDI); 63 received closed head injury instructions (31 SDI, 32 VDI); 45 received upper respiratory infection instructions (18 SDI, 27 VDI); and 31 received vaginal bleeding in early pregnancy instructions (22 SDI, 9 VDI). We used *t*-test to compare the survey scores between the SDI and the VDI groups (Figure). We compared the scores separately in each of the five selected discharge diagnoses and set the statistical significance level to 0.01 to adjust for the multiple testing. The mean survey score was 30.1% (SD = 28.2) in the SDI group and 52.3% (SD = 31.7) in the VDI group (*P* < .001).

There were significantly better survey scores after VDIs vs SDIs for the closed head injury (27% SDI vs 46% VDI, *P* = 0.003); upper respiratory infection (28% SDI vs 64% VDI; *P* < 0.0001); and vaginal bleeding in early pregnancy groups (19% SDI vs 69% VDI, *P* < 0.0001). There were no significant differences in survey scores between the splint care (53% SDI vs 66% VDI; *P* = 0.08) and suture care groups (29% SDI vs 31% VDI; *P* = 0.40).

We also collected data regarding patient satisfaction in the pilot phase of the study. The satisfaction questions were used to gauge how engaging and easily understandable the instructions in the video were. The videos were rated on a scale on 1–5 with “1” being not engaging or understandable and “5” being very engaging and understandable. For the three chief complaints used in the pilot study, the average ratings were 4.53 (splint care), 4.26 (suture care), and 4.38 (upper respiratory infection).

## DISCUSSION

The outcomes of this study suggest that VDI, when compared to SDI, significantly improved immediate patient understanding of discharge instructions for closed head injury, upper respiratory infection, and vaginal bleeding in early pregnancy. Several studies have found similar utility in using VDIs as an adjunct to SDIs in the pediatric ED.^8^–^10^ These findings, along with ours, suggest that a multimedia format such as video provides patients with a multidimensional way of learning that does not bias them based on literacy, educational level, or learning style and that they could improve patient understanding.

Of note, no significant difference in knowledge was found in patients who were given splint care or suture care video compared to SDIs. It is unclear why these two groups showed no difference in understanding. It could be related to these topics being more straightforward, and therefore understanding based on SDI and VDI was similar. For splint care, which had high understanding even with the SDI it is possible that there is a baseline knowledge of how to care for a splint in the community, suggesting that patients required less instruction. For suture care, which consistently scored low in both groups it is possible that our screening and piloting with patients failed and our video was still too complicated to improve patient understanding.

Patients also were satisfied with the videos and found them to be useful. Given the importance placed on patient satisfaction, this suggests that VDIs could have other benefits in addition to improved understanding, which could be evaluated in future research.

## LIMITATIONS

There were some limitations to this study that need to be addressed. First, this study used convenience sampling, which can introduce selection or spectrum bias. In addition, this was a single-center study, which may limit its external validity in EDs with different populations or discharge processes. Furthermore, although the VDIs were created based on the SDIs, it is possible that slight content differences may have been present. These minor changes could potentially have contributed to better retention or understanding of the material presented. Lastly, we did not mandate a specific amount of time for nursing to review paper discharge instructions with the patient; we compared the VDIs to the current standard of care, which likely varies between nurses. Without dedicated time and structure for nursing review of paper discharge instructions with patients it is possible the time spent on the instructions was significantly less than that for the VDI. Arguably, this is one of the benefits of VDI: they are novel, standardized, and the patient is more likely to pay attention. It would be beneficial to determine whether these findings could be duplicated in other EDs with a broad range of chief complaints and more specific guidelines for review of paper discharge instructions.

## CONCLUSION

Educating patients on their diagnosis, treatment plan, and management is an extremely important job of providers that is sometimes overlooked in the treatment process. Lack of attention to this step could put patients at higher risk of preventable complications and overall worse outcomes. Video discharge instructions could improve patient understanding of aftercare instructions and improve patient outcomes. With widespread availability of Internet and smartphone use, implementation of VDIs could be easy to implement more broadly. In fact, some companies create such content and have already partnered with electronic health record systems to allow physicians to “prescribe” educational videos to patients through the EHR, emailing the video to the patient. This study shows the efficacy of such discharge instructions and could encourage further development and utilization of VDIs. Future research should evaluate more widespread implementation and long-term patient outcomes. Implementing video discharge instructions can be way to improve patients’ experience in the ED, while simultaneously ensuring a safe discharge process.

## Supplementary Information





## Figures and Tables

**Figure:**
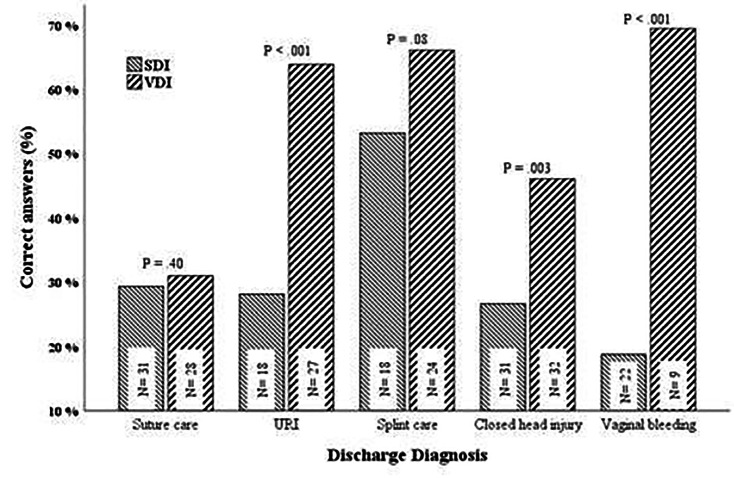
Comparison of scores on patient questionnaires before and after implementation of video discharge instructions. *Denotes significance between standard discharge instruction survey scores and video discharge instruction survey scores (p-value<0.05). *SDI*, standard discharge instructions; *VDI*, video discharge instructions; *URI*, upper respiratory infection.
