# Supplementary material for: Utilization of Educational Videos to Improve Communication and Discharge Instructions
Source: West J Emerg Med. 2021 Apr 27;22(3):644–7. doi: 10.5811/westjem.2021.1.48968 (PMC8202987; doi:10.5811/westjem.2021.1.48968)
Supplement: Supplementary file 1 [file wjem-22-644-s001.docx]

Supplemental File A. Discharge Instruction Questionnaires

Table of Contents:

[Vaginal Bleeding in Early Pregnancy – Questionnaire 2](#_Toc50456456)

[Sangrado vaginal a comienzos del embarazo – Cuestionario 3](#_Toc50456457)

[Vaginal Bleeding in Early Pregnancy – Questionnaire Answers 4](#_Toc50456458)

[Closed Head Injury – Questionnaire 5](#_Toc50456459)

[Lesión cerrada de la cabeza - Cuestionario 7](#_Toc50456460)

[Closed Head Injury – Questionnaire Answers 9](#_Toc50456461)

[Suture Care – Questionnaire 11](#_Toc50456462)

[Cuidado de Suturas (Puntadas) – Cuestionario 12](#_Toc50456463)

[Suture Care – Questionnaire Answers 13](#_Toc50456464)

[Splint Care – Questionnaire 14](#_Toc50456465)

[Cuidado de la férula – Cuestionario 15](#_Toc50456466)

[Splint Care – Questionnaire Answers 16](#_Toc50456467)

[Upper Respiratory Infections – Questionnaire 17](#_Toc50456468)

[Infecciones respiratorias superiores—Cuestionario 18](#_Toc50456469)

[Upper Respiratory Infections – Questionnaire Answers 19](#_Toc50456470)

# Vaginal Bleeding in Early Pregnancy – Questionnaire

**Name: __________________ Length of Stay: _________________**

1. Vaginal bleeding during pregnancy is:

a) Rare

b) Common and often stops on its own

c) Common and always means there is a problem

2. If your doctors were unable to confirm a normal pregnancy on ultrasound you must follow-up for repeat pregnancy hormone (beta-hcg) in:

a) 24 hours

b) 48 hours

c) 3-5 days

3. Which of the following is a cause of a miscarriage?

a) Exercise

b) Nature’s way of ending a pregnancy that was not developing properly

c) Morning sickness

4. Until you follow-up with your doctor you should avoid:

a) Tampons

b) Douching

c) Sexual Intercourse

d) Orgasms

e) All of the above

5. Which of the following symptoms require that you come back to the emergency department or call your doctor? (select all that apply)

a) If the bleeding stops

b) Bleeding through more than two large overnight pads in two hours

c) Fever

d) Fainting episodes

e) Fatigue

f) Chills

h) Leaking fluid or a gush of fluid from your vagina

# Sangrado vaginal a comienzos del embarazo – Cuestionario

**Nombre: __________________ Duración de la estancia: _______________**

1. El sangrado vaginal durante el embarazo es:

a) Raro

b) Común y a menudo se detiene por sí solo

c) Común y siempre significa que hay un problema

2. Si sus médicos no pudieron confirmar un embarazo normal con un ultrasonido debe hacer un seguimiento de la repetición de la hormona del embarazo (beta-hcg) en:

a) 24 horas

b) 48 horas

c) 3-5 días

3. ¿Cuál de las siguientes es una causa de una pérdida de embarazo?

1. Ejercicio
2. La manera de la naturaleza de poner fin a un embarazo que no se estaba desarrollando adecuadamente
3. Nauseas del embarazo

4. Hasta que haga un seguimiento con su médico usted debe evitar:

a) Tampones

b) Duchas vaginales

c) Relaciones sexuales

d) Orgasmos

e) Todo lo anterior

5. ¿Cuáles de los siguientes síntomas requieren que regrese al Departamento de Emergencia o llame a su médico? (seleccionar todo lo que aplique)

a) Si el sangrado se detiene

b) El sangrado empapa dos o más toallas sanitarias nocturnas grandes en dos horas

c) Fiebre

d) Desmayo

e) Fatiga

f) Escalofríos

h) fuga de líquido o un torrente de líquido de la vagina

# Vaginal Bleeding in Early Pregnancy – Questionnaire Answers

**Name: __________________ Length of Stay: _________________**

1. Vaginal bleeding during pregnancy is:

a) Rare

**b) Common and often stops on its own**

c) Common and always means there is a problem

2. If your doctors were unable to confirm a normal pregnancy on ultrasound you must follow-up for repeat pregnancy hormone (beta-hcg) in:

a) 24 hours

**b) 48 hours**

c) 3-5 days

3. Which of the following is a cause of a miscarriage?

a) Exercise

**b) Nature’s way of ending a pregnancy that was not developing properly**

c) Morning sickness

4. Until you follow-up with your doctor you should avoid:

a) Tampons

b) Douching

c) Sexual Intercourse

d) Orgasms

**e) All of the above**

5. Which of the following symptoms require that you come back to the emergency department or call your doctor? (select all that apply)

a) If the bleeding stops

**b) Bleeding through more than two large overnight pads in two hours**

**c) Fever**

**d) Fainting episodes**

e) Fatigue

**f) Chills**

**h) Leaking fluid or a gush of fluid from your vagina**

# Closed Head Injury – Questionnaire

**Name: __________________ Length of Stay: _________________**

1. Which of the following are common and expected symptoms of concussion? (please select all that apply):

a) Mild to moderate headaches

b) Chest pain

c) Difficulty learning or remembering things

d) Difficulty focusing

e) Trouble breathing

f) Neck pain

g) Difficulty making decisions and solving problems

h) Slowness in thinking, acting and reading

i) Feeling tired

j) Passing out or fainting

k) Changes in sleeping habits

l) Feeling dizzy

m) Nausea

n) Weakness or numbness of your extremities

2. What kind of medications can you take for your symptoms? (please select all that apply):

a) Tylenol and Motrin

b) My friend’s pain medications

c) Prescription medications given to me by my doctor

3. What should you do to make your concussion better? (please select all that apply)

a) Play contact sports

b) Rest

c) Avoid strenuous work

d) Avoid watching TV

e) Avoid working on a computer, tablet or smartphone

3. You should return to your activities:

a) As soon as possible

b) Slowly, not all at once

c) In 1 week

4. Which of the following could cause severe injury, brain damage or death:

a) Watching TV

b) Repeat head injury due to contact sports

c) Alcohol

5. Which of the following symptoms require that you come back to the emergency department or call your doctor? (select all that apply)

a) Significant confusion

b) Lots of energy

c) Significant drowsiness

d) Nausea

e) Vomiting

f) Severe headaches not relieved by medicine

g) Weakness or numbness in your arms and legs

h) Inability to walk

i) Tiredness

# Lesión cerrada de la cabeza - Cuestionario

**Nombre: __________________ Duración de la estancia: _______________**

1. ¿Cuáles de los siguientes son síntomas comunes y esperados de una concusión? (por favor seleccionar todo lo que aplique):

a) Dolor de cabeza leve a moderado

b) Dolor de pecho

c) Dificultad para aprender o recordar cosas

d) Dificultad para enfocarse

e) Dificultad para respirar

f) Dolor de cuello

g) Dificultad para tomar decisiones y resolver problemas

h) Lentitud para pensar, actuar o leer

i) Sensación de cansancio

j) Desmayo

k) Cambios en los hábitos de sueño

l) Sentirse mareado

m) Náusea

n) Debilidad o entumecimiento de las extremidades

2. ¿Qué tipo de medicamentos puede tomar para sus síntomas? (por favor seleccionar todo lo que aplique):

a) Tylenol® y Motrin®

b) Medicamentos para el dolor de mi amigo(a)

c) Medicamentos recetados que me ha dado mi médico

3. ¿Qué debe hacer para mejorar su concusión? (por favor seleccionar todo lo que aplique):

a) Jugar deportes de contacto

b) Descansar

c) Evitar el trabajo extenuante

d) Evitar ver la televisión

e) Evitar trabajar en una computadora, tableta o teléfono inteligente

3. Usted debe volver a sus actividades:

a) Tan pronto como sea posible

b) Lentamente, no todo a la vez

c) En 1 semana

4. ¿Cuál de los siguientes podría causar lesiones graves, daño cerebral o la muerte?:

a) Mirar Televisión

b) Repetir lesiones en la cabeza debido a deportes de contacto

c) Bebidas Alcohólicas

5. ¿Cuáles de los siguientes síntomas requieren que regrese al Departamento de Emergencia o llame a su médico? (seleccionar todo lo que aplique)

a) Confusión significativa

b) Mucha energía

c) Somnolencia significativa

d) Náusea

e) Vómito

f) Dolores de cabeza severos no aliviados por la medicina

g) Debilidad o entumecimiento de sus brazos y piernas

h) Incapacidad para caminar

i) Cansancio

# Closed Head Injury – Questionnaire Answers

**Name: __________________ Length of Stay: _________________**

1. Which of the following are common and expected symptoms of concussion? (please select all that apply):

**a) Mild to moderate headaches**

b) Chest pain

**c) Difficulty learning or remembering things**

**d) Difficulty focusing**

e) Trouble breathing

f) Neck pain

**g) Difficulty making decisions and solving problems**

**h) Slowness in thinking, acting and reading**

**i) Feeling tired**

j) Passing out or fainting

**k) Changes in sleeping habits**

**l) Feeling dizzy**

**m) Nausea**

n) Weakness or numbness of your extremities

2. What kind of medications can you take for your symptoms? (please select all that apply):

**a) Tylenol and Motrin**

b) My friend’s pain medications

**c) Prescription medications given to me by my doctor**

3. What should you do to make your concussion better? (please select all that apply)

a) Play contact sports

**b) Rest**

**c) Avoid strenuous work**

**d) Avoid watching TV**

**e) Avoid working on a computer, tablet or smartphone**

3. You should return to your activities:

a) As soon as possible

**b) Slowly, not all at once**

c) In 1 week

4. Which of the following could cause severe injury, brain damage or death:

a) Watching TV

**b) Repeat head injury due to contact sports**

c) Alcohol

5. Which of the following symptoms require that you come back to the emergency department or call your doctor? (select all that apply)

**a) Significant confusion**

b) Lots of energy

**c) Significant drowsiness**

d) Nausea

**e) Vomiting**

**f) Severe headaches not relieved by medicine**

**g) Weakness or numbness in your arms and legs**

**h) Inability to walk**

i) Tiredness

# Suture Care – Questionnaire

**Name: __________________ Length of Stay: _________________**

1. If your wound was closed with staples they should be removed by a healthcare provider in:

a) 1-3 days

b) 4-6 days

c) 7-10 days

d) 11-14 days

2. How do you know when to have your stitches removed?

a) All stitches should be removed in 3 days

b) All stitches should be removed in 14 days

c) Ask your healthcare provider

3. How often should you clean your wound?

a) At least once per day

b) At least once per week

c) Twice per day

d) Every other day

3. Which of the following are signs of an **infection**: (select all that apply)

a) Swelling

b) Redness

c) A scab

d) Pain

e) Fevers

f) Clear fluid or bleeding

g) Vomiting

h) Thick yellow or white pus

4. Which of the following symptoms require that you come back to the emergency department or call your doctor? (select all that apply)

a) Fatigue

b) Signs of infection

c) Bleeding

d) Clear fluid draining from wound

e) Opening of the wound

# Cuidado de Suturas (Puntadas) – Cuestionario

**Nombre: __________________ Duración de la estancia: __________**

1. Si su herida se cerró con grapas, deben ser retiradas por un profesional de la salud en:

a) 1-3 días

b) 4-6 días

c) 7-10 días

d) 11-14 días

2. ¿Cómo sabe cuándo quitarse las suturas/puntadas?

a) Todas las suturas deben retirarse en 3 días

b) Todas las suturas deben retirarse en 14 días

c) Pregunte a su profesional de la salud

3. ¿Con qué frecuencia debe limpiar su herida?

a) Por lo menos una vez al día

b) Al menos una vez por semana

c) Dos veces al día

d) Un día sí y otro no

3. ¿Cuáles de los siguientes son signos de una **infección**?: (seleccionar todo lo que aplique)

a) Hinchazón

b) Enrojecimiento

c) Una costra

d) Dolor

e) Fiebre

f) Líquido transparente o sangrado

g) Vómito

h) Pus espesa amarilla o blanca

4. ¿Cuál de los siguientes síntomas requieren que regrese al Departamento de Emergencia o llame a su médico? (seleccionar todo lo que aplique)

a) Fatiga

b) Signos de infección

c) Sangrado

d) Líquido transparente que drena de la herida

e) Abertura de la herida

# Suture Care – Questionnaire Answers

**Name: __________________ Length of Stay: _________________**

1. If your wound was closed with staples they should be removed by a healthcare provider in:

a) 1-3 days

b) 4-6 days

**c) 7-10 days**

d) 11-14 days

2. How do you know when to have your stitches removed?

a) All stitches should be removed in 3 days

b) All stitches should be removed in 14 days

**c) Ask your healthcare provider**

3. How often should you clean your wound?

**a) At least once per day**

b) At least once per week

c) Twice per day

d) Every other day

3. Which of the following are signs of an **infection**: (select all that apply)

**a) Swelling**

**b) Redness**

c) A scab

**d) Pain**

**e) Fevers**

f) Clear fluid or bleeding

**g) Vomiting**

**h) Thick yellow or white pus**

4. Which of the following symptoms require that you come back to the emergency department or call your doctor? (select all that apply)

a) Fatigue

**b) Signs of infection**

**c) Bleeding**

d) Clear fluid draining from wound

**e) Opening of the wound**

# Splint Care – Questionnaire

**Name: __________________ Length of Stay: _________________**

1. The purpose of a splint is to (select all that apply):

a) Keep bones from moving while they heal

b) Prevent you from itching your injury

c) Be easily removed by you at home

d) Allow swelling after the injury

2. Which of the following should be AVOIDED (select all that apply):

a) Removing the splint at home

b) Sticking pencils and hangars inside to scratch an itch

c) Keeping your injured limb elevated

d) Using your injured limb

e) Placing weight on the splint

f) Getting the splint wet

g) Taking pain medication for pain

3. What should you do before showering with the splint on:

a) Nothing – its waterproof

b) Wrap the splint with a plastic bag to keep it dry

c) Use blow dryer to help it keep its shape when wet

3. Which of the following symptoms require that you come back to the emergency department or call your doctor? (select all that apply)

a) Worsening pain

b) Numbness in your injured limb

c) Blue or white discoloration of your fingers or toes

d) Damaged splint

e) Itching in the splint

f) Splint is too tight or too loose

g) Splint gets wet

Cuidado de la férula – Cuestionario

**Nombre: __________________ Duración de la estancia: __________**

1. El propósito de una férula es (seleccionar todo lo que aplique):

a) Evitar que los huesos se muevan mientras sanan

b) Evitar que le dé comezón en la lesión

c) Se la puede quitar fácilmente en casa

d) Permitir la hinchazón después de la lesión

2. Cuál de los siguientes se debe EVITAR (seleccionar todo lo que aplique):

a) Quitar la férula en casa

b) Introducir lápices y perchas/ganchos dentro para rascar una comezón

c) Mantener su extremidad lesionada elevada

d) Usar la extremidad lesionada

e) Poner peso sobre la férula

f) Mojar la férula

g) Tomar analgésicos para el dolor

3. ¿Qué debe hacer antes de ducharse cuando tiene una férula:

a) Nada – es impermeable

b) Envolver la férula con una bolsa de plástico para mantenerla seca

c) Utilizar un secador para ayudar a mantener su forma cuando se moje

4. ¿Cuáles de los siguientes síntomas requieren que regrese al Departamento de Emergencias o llame a su médico? (seleccionar todo lo que aplique):

a) Dolor que empeora

b) Entumecimiento en la extremidad lesionada

c) Decoloración morada o blanca en los dedos de las manos o los pies

d) Férula dañada

e) Comezón en la férula

f) La férula está demasiado apretada o demasiado suelta

g) La férula se moja

# Splint Care – Questionnaire Answers

**Name: __________________ Length of Stay: _________________**

1. The purpose of a splint is to (select all that apply):

**a) Keep bones from moving while they heal**

b) Prevent you from itching your injury

c) Be easily removed by you at home

**d) Allow swelling after the injury**

2. Which of the following should be AVOIDED (select all that apply):

**a) Removing the splint at home**

**b) Sticking pencils and hangars inside to scratch an itch**

c) Keeping your injured limb elevated

**d) Using your injured limb**

**e) Placing weight on the splint**

**f) Getting the splint wet**

g) Taking pain medication for pain

3. What should you do before showering with the splint on:

a) Nothing – its waterproof

**b) Wrap the splint with a plastic bag to keep it dry**

c) Use blow dryer to help it keep its shape when wet

3. Which of the following symptoms require that you come back to the emergency department or call your doctor? (select all that apply)

**a) Worsening pain**

**b) Numbness in your injured limb**

**c) Blue or white discoloration of your fingers or toes**

**d) Damaged splint**

e) Itching in the splint

**f) Splint is too tight or too loose**

**g) Splint gets wet**

# Upper Respiratory Infections – Questionnaire

**Name: __________________ Length of Stay: _________________**

1. What causes an upper respiratory infection?

a) Bacteria

b) A virus

c) Cold weather

2. What kind of symptoms should you expect as part of your cold? (Select all that apply)

a) Cough

b) Changes in vision

c) Runny nose

d) Nasal congestion

e) Sore throat

f) Blood in urine

g) Fever

h) Headaches

j) Body aches

i) Decreased appetite

3. How long should your symptoms last?

a) 1-3 days

b) 4-6 days

c) 1-2 weeks

4. What can you do to make yourself feel better? (select all that apply)

a) Drink plenty of fluids

b) Take antibiotics

c) Take over the counter Tylenol and Motrin

d) Get plenty of rest

5. Which of the following symptoms require that you come back to the emergency department or call your doctor? (select all that apply)

a) Difficulty breathing

b) Fatigue

c) Coughing up brown or red blood

d) If you feel worse despite following recommendations

e) Vomiting

f) Headaches

g) Body aches

# Infecciones respiratorias superiores—Cuestionario

**Nombre: __________________ Duración de la estancia: __________**

1. ¿Qué causa una infección respiratoria superior?

a) Bacteria

b) Un virus

c) Clima frio

2. ¿Qué tipo de síntomas debe usted esperar como parte de su resfriado? (Seleccionar todo lo que aplique)

a) Tos

b) Cambios en la visión

c) flujo nasal

d) Congestión nasal

e) Dolor de garganta

f) Sangre en la orina

g) Fiebre

h) Dolores de cabeza

j) Dolor en el cuerpo

i) Disminución del apetito

3. ¿Cuánto tiempo deben durar los síntomas?

a) 1-3 días

b) 4-6 días

c) 1-2 semanas

4. ¿Qué puede hacer para sentirse mejor? (seleccionar todo lo que aplique)

a) Beber suficiente líquidos

b) Tomar antibióticos

c) Tomar Tylenol® y Motrin® que puede obtener sin receta

d) Descansar bastante

5. ¿Cuál de los siguientes servicios requieren que regrese al Departamento de Emergencia o llame a su médico? (seleccionar todo lo que aplique)

a) Dificultad para respirar

b) Fatiga

c) Tos con sangre marrón o roja

d) Si se siente peor a pesar de seguir las recomendaciones

e) Vómito

f) Dolores de cabeza

g) Dolor en el cuerpo

# Upper Respiratory Infections – Questionnaire Answers

**Name: __________________ Length of Stay: _________________**

1. What causes an upper respiratory infection?

a) Bacteria

**b) A virus**

c) Cold weather

2. What kind of symptoms should you expect as part of your cold? (Select all that apply)

**a) Cough**

b) Changes in vision

**c) Runny nose**

**d) Nasal congestion**

**e) Sore throat**

f) Blood in urine

**g) Fever**

**h) Headaches**

**j) Body aches**

**i) Decreased appetite**

3. How long should your symptoms last?

a) 1-3 days

b) 4-6 days

**c) 1-2 weeks**

4. What can you do to make yourself feel better? (select all that apply)

**a) Drink plenty of fluids**

b) Take antibiotics

**c) Take over the counter Tylenol and Motrin**

**d) Get plenty of rest**

5. Which of the following symptoms require that you come back to the emergency department or call your doctor? (select all that apply)

**a) Difficulty breathing**

b) Fatigue

**c) Coughing up brown or red blood**

**d) If you feel worse despite following recommendations**

e) Vomiting

f) Headaches

g) Body aches
