# Supplementary material for: Utilization of Educational Videos to Improve Communication and Discharge Instructions
Source: West J Emerg Med. 2021 Apr 27;22(3):644–7. doi: 10.5811/westjem.2021.1.48968 (PMC8202987; doi:10.5811/westjem.2021.1.48968)
Supplement: Supplementary file 2 [file wjem-22-644-s002.docx]

Supplemental File B: Discharge Instruction Video Links

Vaginal Bleeding in Early Pregnancy – Video Link

- <https://youtu.be/BESNIzltKqI>

Sangrado vaginal a comienzos del embarazo – Video Link

- <https://youtu.be/BIw8VrwHlLs>

Closed Head Injury – Video Link

- <https://youtu.be/Phn3XU-6vo8>

Lesión cerrada de la cabeza—Video Link

- <https://youtu.be/f_xRHZ-5l6k>

Suture Care – Video Link

- <https://youtu.be/gHrxkl9QBhQ>

Cuidado de Suturas (Puntadas)—Video Link

- <https://youtu.be/hVPxU3pufLY>

Splint Care – Video Link

- <https://youtu.be/-gdA-6zcpjY>

Cuidado de la férula—Video Link

- <https://youtu.be/8YRZUa2aUwo>

Upper Respiratory Infections – Video Link

- <https://youtu.be/aW_ugQaLp28>

Infecciones respiratorias superiores— Video Link

- <https://youtu.be/_gI7dCnHjDk>
